# “I don’t want to be a burden”: the double-edged role of filial piety in fall-risk perception among community-dwelling older adults in China

**DOI:** 10.3389/fpubh.2026.1875906

**Published:** 2026-07-21

**Authors:** Yidan Zhu, Dan Zhang, Yan Deng, Yu Xiong

**Affiliations:** 1 School of Nursing, Hunan Normal University, Changsha, China; 2 Hunan Cancer Hospital, Changsha, China

**Keywords:** accidental falls, China, community-dwelling older adults, fall prevention, fall-risk perception, filial piety, healthy aging, qualitative research

## Abstract

**Background:**

Falls are a major threat to healthy aging, yet older adults’ subjective appraisal of their likelihood and consequences of falling may differ from objective physiological risk. Fall-risk perception is broader than fear of falling or activity-specific concern and is relevant because it may inform older adults’ willingness to disclose falls, seek help, modify the home environment, and participate in balance or strength training.

**Aim:**

This study aimed to explore how community-dwelling older adults in China experience and understand fall risk in everyday life and family relationships, with particular attention to filial piety and the desire not to become a burden to adult children.

**Methods:**

A descriptive phenomenological design was used. Nineteen community-dwelling older adults in Changsha, China, were purposively recruited through maximum variation sampling and stratified by levels of concern about falling assessed using the Falls Efficacy Scale-International (FES-I). The FES-I was used as a sampling aid rather than as an operational measure of the broader phenomenon of fall-risk perception. Semi-structured individual interviews were conducted between March and May 2023. Data were analyzed using Colaizzi’s seven-step method. Reporting followed the Consolidated Criteria for Reporting Qualitative Research.

**Results:**

Three overarching themes were identified. First, embodied and relational experiences were associated with heightened fall-risk perception, including adverse fall-related events, perceived efficacy of prevention, awareness of physical decline, environmental vigilance, and caregiver monitoring. Second, cognitive, identity-related, and contextual experiences were associated with reduced risk appraisal or constrained prevention, including misconceptions about falls, normalization of “harmless” falls, denial and overconfidence, over-reliance on assistive devices, living circumstances limiting home-safety practices, and limited professional support. Third, filial responsibility appeared in two contrasting forms: the wish not to burden adult children was associated with greater caution, while some participants also concealed minor falls to avoid causing family worry.

**Conclusion:**

Participants’ accounts suggest that fall-risk perception among community-dwelling older adults in China is experienced not only as an individual appraisal but also within family and cultural relationships. Community nurses should consider integrating objective fall-risk assessment with family-sensitive communication strategies that promote mobility, autonomy, and family well-being while minimizing guilt, stigma, and concealment.

## 1 Introduction

Falls represent a major public health challenge and disproportionately affect the health, independence, and longevity of older adults. Global guidelines emphasize that fall prevention requires assessment of modifiable risks and interventions that include exercise, medication review, environmental modification, vision optimization, and individualized multifactorial management (1, 2). In China, rapid population aging has intensified the burden of falls. National data show that falls are a major cause of injury-related mortality among older adults, and the disease burden attributable to falls has increased substantially over recent decades (3, 4).

Evidence supports the effectiveness of fall-prevention interventions, especially supervised balance and resistance training, Tai Chi, high-dose education or cognitive-behavioral approaches, and multifactorial assessment with targeted treatment (5–7). However, effectiveness in real-world community settings depends not only on the availability of interventions but also on whether older adults recognize their own risk and are willing to act (8–10). Fall-risk perception, broadly defined as a person’s subjective appraisal of the likelihood and potential consequences of falling, is therefore relevant to preventive behaviors such as home modification, appropriate use of assistive devices, help-seeking, and participation in strength and balance training (11–13).

In this study, fall-risk perception was used as an umbrella construct comprising perceived susceptibility, anticipated severity, perceived controllability, and the personal and relational meaning of falling. It differs from perceived susceptibility, which concerns the judged likelihood of a fall; fear of falling, which is primarily an affective response; fall efficacy, which refers to confidence in performing activities without falling; and concern about falling during activities, which is commonly assessed with the Falls Efficacy Scale-International (14). Fall-risk awareness refers more specifically to recognition of fall hazards and the degree to which subjective appraisal corresponds with objective risk. Although these constructs are related and may coexist, they are not interchangeable (11–13, 15–19). Consistent with the phenomenological orientation, the present study examined how participants experienced, interpreted, and communicated fall risk rather than treating it as a single measurable variable.

These distinctions are clinically relevant because a persistent mismatch may exist between perceived and physiological fall risk. Some older adults with high objective risk underestimate their vulnerability, whereas others experience excessive fear of falling that restricts activity and accelerates deconditioning (15–17). Recent reviews have shown that older adults often lack accurate knowledge of fall risk factors, do not always see themselves as personally at risk, and may perceive fall-prevention information as irrelevant, repetitive, or stigmatizing (10, 19). These findings suggest that fall-risk perception cannot be reduced to knowledge alone; it also involves emotion, identity, life experience, perceived control, and social context.

Qualitative evidence further indicates that the language of “falls,” “fall prevention,” “old age,” and “frailty” may threaten older adults’ identities. Van Scherpenseel et al. found that community-dwelling older adults preferred positive reframing and messages emphasizing “staying fit and healthy” or “living independently for longer” rather than confronting labels related to aging and falling (20). This insight is important for nursing practice because older adults may resist prevention when it implies that they are frail, dependent, or “the type of person who falls”.

Most existing literature has been generated in Western settings, where concerns about falls are often framed around independence, autonomy, and fear of institutionalization (17, 18). In Confucian-heritage societies such as China, however, later-life health behavior is also embedded in family obligation, intergenerational reciprocity, and filial piety (21, 22). Filial piety has been conceptualized as a multidimensional ethical and relational norm that structures intergenerational care through respect, obedience, material assistance, emotional support, and the maintenance of family harmony (22). Existing evidence indicates that filial norms and intergenerational support can shape older adults’ health-care seeking and willingness to accept assistance, while also influencing family caregiving expectations and perceptions of burden (21–23). In the context of falls, these norms may have ambivalent implications. On the one hand, older adults may become more cautious because a fall could increase caregiving demands for adult children. On the other hand, concern about troubling children may discourage disclosure of minor falls, requests for environmental modification, or professional help-seeking. However, the role of filial piety in older adults’ fall-risk perception remains underexplored, particularly with regard to risk appraisal, disclosure, and help-seeking within everyday family relationships. Addressing this gap is important for developing culturally sensitive fall-prevention strategies in Chinese community settings.

Although Chinese scholarship has begun to emphasize fall-risk perception as a basis for active fall prevention, the conceptual connotation of fall-risk perception remains underdeveloped, and empirical evidence on how older adults construct risk in daily life is still limited (12, 24, 25). Therefore, this descriptive phenomenological study aimed to explore the lived experience of fall-risk perception among community-dwelling older adults in China. Specifically, we explored how embodied, cognitive, environmental, professional, and family-related experiences shape older adults’ perceptions of fall risk within the Chinese cultural context.

## 2 Methods

### 2.1 Study design and methodology

This study employed a Husserlian descriptive phenomenological design, which aims to describe the essential structure and meanings of lived experience while suspending, as far as possible, pre-existing theoretical and clinical assumptions (26). Descriptive phenomenology was selected rather than interpretive phenomenology because the study sought a faithful description of how older adults experienced and articulated fall risk, rather than a hermeneutic interpretation of historically situated meanings or the development of a causal theory. The approach was operationalized through open-ended interviewing, close attention to participants’ own language, ongoing bracketing, and Colaizzi’s movement from significant statements to formulated meanings and an exhaustive description (27). The working definition of fall-risk perception bounded the phenomenon of interest but did not impose a predetermined coding model. Reporting followed the Consolidated Criteria for Reporting Qualitative Research (28).

### 2.2 Participants and sampling

Purposive sampling with maximum variation was used to recruit community-dwelling older adults from multiple communities in Changsha, China, between March and May 2023. Inclusion criteria were: (1) age ≥60 years; and (2) ability to communicate and provide informed consent. Exclusion criteria were: (1) being bedridden for more than 30 consecutive days; and (2) severe cognitive impairment or psychiatric disorders. The maximum-variation criteria were specified prospectively and used to guide participant selection, rather than being recorded solely as descriptive characteristics. Variation was sought in fall-related concern, sex, age, educational level, living arrangement, and fall history. To ensure diversity in fall-related concern, we administered the Falls Efficacy Scale-International (FES-I) before interview scheduling and stratified participants into three groups using established cutoffs: low concern (16–19), moderate concern (20–27), and high concern (28–64), with at least three participants in each group (29). Sample size was determined by data saturation, defined as the point at which no new themes or subthemes emerged from three consecutive interviews (Table 1).

**Table 1:** Sample characteristics (*N* = 19).

*N* = 19	Sex	Age(years)	Educational level	Living arrangement	Fall history
N1	Male	74	Primary school	With spouse	No
N2	Female	78	High school	Alone	Yes
N3	Male	64	Undergraduate	With spouse	No
N4	Female	72	Junior high school	Alone	No
N5	Female	87	Primary school	With daughter	Yes
N6	Female	76	Junior high school	With son	Yes
N7	Male	64	High school	With spouse	No
N8	Female	73	Junior high school	Alone	Yes
N9	Male	75	Primary school	Alone	No
N10	Female	80	Primary school	With spouse	Yes
N11	Female	74	High school	With spouse	Yes
N12	Male	66	Junior high school	With spouse	No
N13	Male	68	Undergraduate	With spouse	No
N14	Female	83	Primary school	With son	Yes
N15	Male	75	Junior high school	With spouse	No
N16	Female	82	Primary school	With son	Yes
N17	Female	73	Junior high school	With spouse	Yes
N18	Male	77	High school	With son	Yes
N19	Male	63	Junior high school	With spouse	No

### 2.3 Researcher characteristics and reflexivity

The first author, a female nursing graduate student, conducted all interviews. She had received systematic training in qualitative research and had no prior relationship with participants. To minimize social desirability bias, she emphasized confidentiality, clarified that there were no right or wrong answers, and adopted a non-judgmental stance. Bracketing was approached as an ongoing reflexive practice rather than a single event. The first author maintained a reflexive journal throughout recruitment, interviewing, transcription, and analysis, recording expectations, emotional responses, and emerging interpretations. Specific preconceptions included the assumptions that older adults would naturally disclose falls to family members and would welcome professional prevention advice. Reflexive notes were reviewed during research team meetings alongside verbatim excerpts to make these assumptions visible and to distinguish participant-grounded meanings from prior nursing expectations. Independent coding of three transcripts and discussion among all four researchers provided an additional check against premature interpretive closure. Bracketing was used to limit the unexamined influence of prior assumptions rather than to claim complete researcher neutrality. The research team included a nursing professor with extensive experience in geriatric qualitative research, who supervised the analysis.

### 2.4 Data collection

Each participant completed one data-generating individual interview; no repeat data-collection interviews were conducted. A semi-structured interview guide was developed based on a literature review and pilot tested with two older adults. The two pilot interviews were included in the final analysis because no substantive changes were made to the interview guide after pilot testing. The final guide included open-ended questions such as: “Why do you think you are or are not at risk of falling?”; “What do you do to prevent falls?”; “What would a fall mean for your life and your family?”; “Would you tell your children if you had a minor fall?”; and “What kind of support from family or professionals would make fall prevention easier?” These family-related questions were included to explore how intergenerational relationships and perceived burden shaped risk appraisal and disclosure. Interviews were conducted between March and May 2023 in private settings chosen by participants, such as rooms in community health centers, and lasted 40–60 min. All interviews were audio-recorded after written informed consent was obtained. Field notes documenting non-verbal cues, contextual details, and preliminary analytic impressions were recorded immediately after each interview.

### 2.5 Data analysis

Audio recordings were transcribed verbatim within 24 h by the first author and checked for accuracy against the original recordings by the second author. Transcripts were entered into NVivo 12 for data management. Data collection and preliminary analysis proceeded iteratively. After each interview, the first author reviewed the transcript and field notes against the evolving list of significant statements, codes, and provisional themes. Preliminary findings were discussed regularly among all four researchers. Saturation was monitored using a prospective saturation log, which documented whether each interview generated new codes, formulated meanings, or changes to the thematic structure. The saturation log and emerging coding framework were reviewed by all four researchers. Recruitment ended after three consecutive interviews yielded no substantive additions, and the research team agreed that these interviews constituted a confirmation set. Data were analyzed using Colaizzi’s seven-step method (27): (1) reading the transcripts repeatedly to gain overall familiarity; (2) extracting significant statements; (3) formulating meanings; (4) clustering meanings into themes; (5) integrating the themes into a comprehensive description; (6) validating the description with participants; and (7) incorporating participant feedback into the final report. The first and second authors independently coded three transcripts and met to compare codes and refine the coding framework. The third and fourth authors subsequently reviewed the coding framework, formulated meanings, and developing thematic structure. Any discrepancies were discussed by all four researchers until consensus was reached. All four researchers reviewed and approved the final themes and subthemes.

### 2.6 Methodological trustworthiness

Methodological trustworthiness was considered according to Lincoln and Guba’s criteria of credibility, transferability, dependability, and confirmability (30). Credibility was supported through maximum-variation sampling, iterative data collection and analysis, member checking of the comprehensive description, and regular discussion of preliminary findings within the research team. Transferability was enhanced by providing detailed descriptions of the study setting, participant characteristics, sampling strategy, interview procedures, and cultural context. Dependability was supported by maintaining an audit trail that included the interview guide, field notes, reflexive journals, coding decisions, the evolving thematic framework, and the prospective saturation log. Confirmability was strengthened through ongoing bracketing, independent coding of selected transcripts, comparison of interpretations against verbatim quotations, and consensus discussions among all four researchers. These strategies were used to ensure that the findings remained grounded in participants’ accounts rather than in the researchers’ prior clinical assumptions.

### 2.7 Ethical considerations

This study was approved by the Biomedical Research Ethics Committee of Hunan Normal University (Approval No. 2023–315). All participants provided written informed consent after receiving a full explanation of the study purpose, procedures, audio recording, confidentiality measures, and their right to withdraw at any time without consequences. Interview data were anonymized using participant codes and stored securely by the research team.

## 3 Results

Three overarching themes were identified: (1) embodied and relational experiences associated with heightened fall-risk perception; (2) cognitive, identity-related, and contextual experiences associated with reduced risk appraisal or constrained prevention; and (3) filial responsibility in accounts of caution and concealment. Table 2 summarizes the themes and subthemes.

**Table 2:** Themes and subthemes.

Theme	Subthemes
Embodied and relational experiences associated with heightened fall-risk perception	Wake-up calls from adverse fall events; perceived efficacy of prevention; awareness of physical decline; environmental risk vigilance; caregiver monitoring and reinforcement
Cognitive, identity-related, and contextual experiences associated with reduced risk appraisal or constrained prevention	Misconceptions about falls; normalization of “harmless” falls; denial and overconfidence; over-reliance on assistive devices; living circumstances limiting home-safety practices; limited professional support
Filial responsibility in accounts of caution and concealment	Filial responsibility as a motivator for caution; filial responsibility as a reason for concealment

### 3.1 Embodied and relational experiences associated with heightened fall-risk perception

#### 3.1.1 Wake-up calls from adverse fall events

Participants described personal falls and witnessing others’ falls as powerful experiential cues that made risk tangible. Some participants connected falls with fracture, disability, or death. One participant said, “I’ve been through it. If you fracture a bone, it’s a big problem. You could be gone” (N5, Female, 87). Another participant reflected, “Now that I’ve fallen, I realize how serious it is. It really is necessary to prevent it” (N18, Male, 77). Vicarious experience also functioned as a warning: “I’ve never fallen, but my mother did. She’s had lasting effects ever since” (N3, Male, 64).

#### 3.1.2 Perceived efficacy of prevention

Participants who regarded prevention as actionable tended to acknowledge fall risk more readily. Some trusted environmental adjustments: “I make some changes—wear non-slip shoes, and I try to keep the floor dry at home” (N3, Male, 64). Others relied mainly on personal carefulness: “Being careful—that’s the most effective way. If you are careless, you could fall any day” (N8, Female, 73). Some participants accepted the value of prevention in principle but implemented it inconsistently: “In theory, I should do balance training. But when it’s cold, I just do not go out” (N13, Male, 68).

#### 3.1.3 Awareness of physical decline

Awareness of bodily change made some participants more vigilant. Several described compensatory adaptation through self-monitoring and activity adjustment: “Do not do things you cannot manage. Measure blood pressure daily, take medication—that’s important” (N16, Female, 82). Others acknowledged age-related decline reluctantly: “At our age, falls happen for many reasons. The same situation might not have caused a fall when we were younger, but now it’s hard to say” (N17, Female, 73). For those who had experienced injury, awareness of decline was accompanied by helplessness: “Of course I’m afraid. Look, my hand still is not right. If I fall again, I will not even have a hand to catch myself. I walk very slowly now, knowing my body is not good” (N6, Female, 76).

#### 3.1.4 Environmental risk vigilance

Participants who recognized environmental hazards described avoidance, attentional focus, and careful movement. One participant explained, “On rainy days I stay home. When I’m out, I’m very careful—hold onto things” (N2, Female, 78). Another said, “I pay attention, especially when moving around. I only concentrate on walking, never use my phone. You never know what might happen on the road” (N3, Male, 64). Night-time toileting was experienced as risky because darkness, urgency, and difficulty locating assistive devices overlapped: “I’m a bit scared when I get up at night, no light, sometimes I cannot find my cane. Now I will not move without it” (N11, Female, 74).

#### 3.1.5 Caregiver monitoring and reinforcement

Family caregivers served as monitors and reinforcers of fall-prevention awareness. Adult children transmitted health information, prompted attention to daily safety, and initiated practical protective measures after a fall. One participant stated, “My daughter knows a lot—she sees it online and tells me” (N5, Female, 87). Another participant described how his daughter responded after a fall: “I fell quite badly last time, bled, but did not go to hospital. Afterwards, my daughter bought me a cane. At first, I did not like it, but then I got used to it. It makes me feel safe” (N15, Male, 75). Together, these accounts show that caregiver involvement operated through both informational reminders and concrete reinforcement, making fall risk more visible and supporting prevention in everyday life.

### 3.2 Cognitive, identity-related, and contextual experiences associated with reduced risk appraisal or constrained prevention

#### 3.2.1 Misconceptions about falls

Some participants framed falls as random, unavoidable events, which reduced their sense of personal control. One participant explained, “There are so many reasons—you can fall anywhere. Some can be avoided, but some you just cannot escape” (N16, Female, 82). Others expressed prevention futility: “If you are going to fall and get sick, it’s the same for everyone. Maybe older people recover more slowly, but I’m in good health” (N7, Male, 64). Another participant displaced fall risk onto exceptional accidents: “It will not affect anything—I will not fall. Unless it’s something else, then it’s not just a fall, it’s an accident” (N19, Male, 63).

#### 3.2.2 Normalization of risk due to “harmless” falls

Minor falls or uneventful near-falls led some participants to normalize fall risk. These experiences encouraged the belief that falling was common, manageable, or unlikely to produce serious consequences. One participant stated, “None of my friends have fallen, and even if they did, they’d get up—nothing serious” (N1, Male, 74). Another explained, “I often get tripped by my grandson, but it’s nothing, still healthy” (N12, Male, 66). This normalization undermined the perceived need for systematic prevention: “I fell once, but nothing happened, so if it’s not serious, it’s okay” (N17, Female, 73).

#### 3.2.3 Denial, overconfidence, and resistance to the identity of frailty

Denial and overconfidence were expressed in ways that appeared to preserve a capable self-image while diminishing appraisal of fall risk. Overestimation of ability was common: “No, I will not fall. I know my body—I can work all day without a problem” (N19, Male, 63). Others rejected the identity of being old or frail: “Old? Am I old? You should ask those in their eighties—I’m still far from that” (N7, Male, 64). Some resisted assistive devices because they symbolized decline: “I can walk on my own. A cane would just get in the way, and it’s not nice” (N14, Female, 83).

#### 3.2.4 Over-reliance on assistive devices

Although assistive devices were described as improving confidence, strong reliance on them was sometimes accompanied by a sense of security that exceeded participants’ attention to residual risk. One participant asked rhetorically, “Using a cane should be safe, right?” (N10, Female, 80). Another explained how the cane became a necessary condition for movement: “I’m used to the cane now—if I feel for it before standing up, I’m not afraid” (N11, Female, 74). These accounts suggest that perceived protection from a cane could narrow attention to other situational or environmental hazards.

#### 3.2.5 Living circumstances limiting home-safety practices

Living circumstances shaped both vigilance toward home hazards and participants’ capacity to act on perceived risks. Long-term familiarity with the home sometimes created a sense of safety that reduced attention to potential hazards. One participant said, “How could I fall at home? I’ve lived here for decades—I could walk around with my eyes closed” (N15, Male, 75). Residence in an adult child’s home could limit perceived authority to request environmental changes: “I live in my son’s house; I will not ask for changes. If it were my own place, it’d be different” (N6, Female, 76). Living alone made some potentially risky daily tasks feel unavoidable: “Living alone, if you do not do things, nobody does. You cannot worry about everything” (N2, Female, 78). Together, these accounts indicate that limited home-safety practices reflected a broader configuration of familiarity, perceived control over the dwelling, and the practical demands of living alone or with family, rather than co-residence status alone.

#### 3.2.6 Limited professional support

Participants reported limited access to professional, individualized fall-prevention guidance. Some perceived community education as repetitive and ineffective: “The community always says the same things—useless, I already know” (N9, Male, 75). Others expressed a need for professional instruction: “There are too few activities for older people; we organize them ourselves, with no professional guidance” (N6, Female, 76), and “I have not heard anything—we need professionals to tell us” (N11, Female, 74). Hidden knowledge gaps were also evident: “I do not know how to prevent falls—never heard of it. I guess just be careful” (N15, Male, 75).

### 3.3 Filial responsibility in accounts of caution and concealment

#### 3.3.1 Filial responsibility as a motivator for caution

The desire not to become a burden to adult children was frequently described alongside greater caution. In these accounts, fall prevention was understood not only as self-protection but also as a way to limit additional caregiving demands on children. One participant explained, “I live alone. If I fell and it was serious, someone would need to look after me. My son has to work, so it would trouble them” (N2, Female, 78). Another echoed, “I am careful, I do not push myself. Otherwise I suffer, and my daughter suffers too—she has to work” (N5, Female, 87).

#### 3.3.2 Filial responsibility as a reason for concealment

The same concern was also present in accounts of concealment. Some participants avoided telling their children about minor falls because disclosure might generate worry, criticism, or additional family responsibilities. One participant said, “My daughter has her own family now, I do not want to bother them” (N11, Female, 74). Another stated, “Sometimes when I fall, and it’s not serious, I do not tell them” (N16, Female, 82). Across these accounts, filial responsibility was associated with both caution and the withholding of information that caregivers and health professionals might otherwise use for timely prevention.

## 4 Discussion

### 4.1 Principal findings

The findings suggest that fall-risk perception among community-dwelling older adults in China was experienced not as a static cognitive judgment but as an appraisal embedded in bodily change, identity, everyday environments, and family relationships. Participants described experiential cues, bodily awareness, environmental vigilance, and caregiver monitoring as heightening risk awareness, whereas misconceptions, normalization of minor falls, overconfidence, strong reliance on assistive devices, living circumstances limiting home-safety practices, and limited professional support were associated with reduced appraisal or constrained preventive action. The principal contribution is the finding that participants narrated the same filial concern—the wish not to burden adult children—in two contrasting ways: as a reason for caution and as a reason to conceal minor falls.

### 4.2 Fall-risk perception as identity-protective sense-making

Our findings are consistent with previous evidence that older adults may underestimate their fall risk, lack knowledge of fall-prevention strategies, or perceive prevention as irrelevant when they do not see themselves as personally vulnerable (31–33). The present findings further suggest that denial and overconfidence may not reflect lack of knowledge alone. In participants’ accounts, these responses appeared to preserve a sense of capability and autonomy. This interpretation aligns with research showing that falls can symbolize frailty, disability, and dependency, making fall prevention a potentially stigmatized topic rather than a neutral health message (34).

The findings also resonate with the reframing strategy proposed by van Scherpenseel et al., who found that older adults preferred positively framed communication emphasizing staying fit, healthy, and independent rather than direct references to “fall prevention” or “old age” (20, 35). In the present study, similar identity dynamics were evident when participants distanced themselves from being categorized as old, frail, or at risk. Nurses and community health workers should therefore avoid assuming that warning-oriented education will necessarily increase engagement. Fear-based or stigmatizing communication may contribute to defensiveness or reduced disclosure, particularly among older adults who seek to maintain a capable self-identity.

### 4.3 Cultural specificity: from independence to relational responsibility

Western studies often describe fear of falling as a threat to independence and personal freedom (18). Although independence was relevant in this study, participants frequently framed falls relationally: a serious fall could trouble children, interrupt their work, and increase family caregiving demands. These accounts extend previous research by suggesting that fall-risk perception in Chinese community settings may be organized around relational responsibility as well as individual vulnerability. Participants linked filial concerns with self-monitoring, risk avoidance, disclosure, and help-seeking; however, these descriptive associations should not be interpreted as evidence of causal effects.

This relational pattern should be interpreted cautiously. In participants’ accounts, filial values were associated with family cohesion and preventive caution, but also with guilt and concealment. Family reminders may be experienced as care while simultaneously making older adults feel dependent or morally responsible for reducing their children’s worries. This tension may help explain why some participants appeared receptive to family advice yet concealed minor falls or avoided requesting environmental modifications when they regarded an event as “not serious.” Recent nursing literature describes filial piety as a multidimensional and context-dependent construct associated with family support, caregiving expectations, caregiver burden, and culturally sensitive care needs (22, 23, 36). The present findings contribute the older adult’s perspective: the wish not to burden children was narrated as both a protective consideration and a potential source of hidden risk. In this dataset, filial piety is therefore better understood as a relational context within which responsibility, autonomy, and communication were negotiated, rather than as a single causal mechanism.

### 4.4 Clinical and practical implications

Community nurses should assess fall-risk perception alongside objective fall-risk indicators. In addition to asking whether a fall occurred, nurses can use brief exploratory questions such as: “What would a fall mean for your family?” “Would you tell your children if you had a minor fall?,” “What makes you feel safe or unsafe at home?,” and “What kind of support would help you prevent falls without feeling like a burden?” These questions can reveal cognitive distortions, concealment tendencies, family pressure, and practical barriers that may not emerge through checklist-based screening alone.

Second, nurse-led education should address normalization bias without increasing fear or shame. Rather than presenting fall prevention as a sign of frailty, educational messages can be reframed as maintaining mobility, protecting family routines, and enabling older adults to continue valued roles. Examples include “steady steps for healthy living,” “balance and strength for safe travel,” or “staying active without worrying your family.” Such benefit-oriented and non-stigmatizing framing is consistent with evidence that older adults respond better to messages about health, independence, and meaningful activity than to labels emphasizing falling and decline (20, 35).

Third, family-inclusive interventions may seek to reframe filial responsibility from a source of concealment into a shared preventive partnership. Nurses may guide adult children to offer supportive rather than controlling reminders and to avoid messages that increase guilt, shame, or fear of being a burden. Family sessions could focus on open communication after minor falls, safe home modification, appropriate use of assistive devices, and shared decision-making. Because family caregivers were not interviewed in this study, these recommendations should be evaluated in dyadic or family-centered research before assumptions are made about how adult children understand or enact filial responsibility. Chinese guidance on fall prevention emphasizes adaptation of evidence-based recommendations to local health-care and community contexts, while implementation strategies continue to require refinement (1, 37, 38).

Fourth, home-safety assessment should extend beyond a checklist of physical hazards to the older adult’s broader living circumstances, including familiarity with the dwelling, perceived authority to alter it, co-residence relationships, and unavoidable daily tasks. A qualitative meta-synthesis found that older adults’ home-related decisions reflect autonomy, attachment, safety, aesthetics, and family connection rather than dwelling type alone (39). Chinese qualitative evidence likewise indicates that social support and adjustment of the home environment are central to fall-prevention self-management, while barriers and unmet professional support needs remain common (40). Community nurses should therefore explore who controls household decisions, which modifications are acceptable, and which daily tasks cannot realistically be avoided.

Finally, community programs should move beyond merely delivering repetitive lectures. Participants in this study reported limited professional guidance and a need for individualized instruction. Experiential training, such as supervised balance practice, home-hazard walkthroughs, and demonstrations of safe assistive-device use, may enhance self-efficacy while challenging overconfidence. Nurses are well-positioned to coordinate these programs with community health centers, family caregivers, physical therapists, and neighborhood organizations.

### 4.5 Strengths and limitations

This study used several procedures intended to enhance methodological trustworthiness, including maximum variation sampling, FES-I-based diversification of concern levels, reflexive practice, independent coding of selected transcripts, member checking, and COREQ-guided reporting. It also contributes culturally situated accounts of how filial responsibility was experienced in relation to fall-risk perception. Several limitations should be acknowledged. First, the sample was drawn from one city in China, which may limit transferability to rural areas or regions with different family structures and community resources. Second, interviews relied on retrospective accounts, introducing potential recall bias; future studies could use prospective diaries or ecological momentary assessment to capture real-time appraisal and disclosure decisions. Third, prolonged engagement with participants was not undertaken. Fourth, translation of quotations from Chinese to English may have altered nuances despite bilingual checking. Finally, family caregivers were not interviewed. The analysis therefore reflects older adults’ interpretations of family expectations rather than corroborated dyadic processes and cannot establish adult children’s intentions or causal effects of filial norms on behavior. Future studies should use dyadic or family-centered qualitative designs to examine how older adults and adult children jointly negotiate fall risk, disclosure, responsibility, and preventive action.

## 5 Conclusion

Participants’ accounts illustrate the complexity of fall-risk perception among community-dwelling older adults in China. Adverse fall events, awareness of physical decline, environmental vigilance, perceived prevention efficacy, and caregiver monitoring were described as heightening risk awareness, whereas misconceptions, normalization, overconfidence, strong reliance on assistive devices, living circumstances limiting home-safety practices, and limited professional support were associated with reduced appraisal or restricted preventive action. Filial responsibility was narrated in a double-edged manner: the desire not to burden children accompanied greater caution for some participants but also the concealment of minor falls for others. These findings describe relational meanings rather than causal effects. These findings highlight the importance of culturally sensitive, family-oriented fall-prevention strategies that acknowledge both individual and relational meanings of fall risk.

## Data Availability

The original contributions presented in the study are included in the article/supplementary material, further inquiries can be directed to the corresponding author/s.
